# Retrospective Perceptions of Changes in Well-Being: The Impact of Age, Culture, and Aging Attitudes on Accuracy

**DOI:** 10.1093/geroni/igab046.731

**Published:** 2021-12-17

**Authors:** Jeongsoo Park, Allura Lothary, Thomas Hess

**Affiliations:** 1 Ajou University, Suwon-si, Kyonggi-do, Republic of Korea; 2 University of Illinois Urbana-Champaign, Urbana, Illinois, United States; 3 North Carolina State University, Raleigh, North Carolina, United States

## Abstract

Aging attitudes have important consequences on functioning in later-life. A critical question concerns whether such attitudes may bias perceptions of one’s own aging, with potentially negative effects on important outcomes. Using data from adults aged 30 – 85 in the US (n=315), Hong Kong (n=317), and Germany (n=623), we examined the impact of age and aging attitudes on accuracy of perceptions of change in well-being over five years in different domains of functioning. Across contexts, comparisons revealed good correspondence between retrospective reports and actual change. However, older adults and those with negative attitudes retrospectively reported less positive change over this period. Accuracy of perceived change was affected by aging attitudes, with positive attitudes being associated with greater accuracy across most domains, although culture moderated these effects. The results highlight the complex relationship between culture and perceptions of well-being, as well as the potentially insidious effects of attitudes on their accuracy.

